# Patterns of Attrition in the Canadian Armed Forces Members and Veterans Mental Health Follow-up Survey (CAFVMHS)

**DOI:** 10.1177/07067437211002697

**Published:** 2021-03-19

**Authors:** Shay-Lee Bolton, Tracie O Afifi, Natalie P Mota, Murray W. Enns, Ron de Graaf, Ruth Ann Marrie, Scott B. Patten, Lisa M. Lix, Jitender Sareen

**Affiliations:** 1 Department of Psychiatry, University of Manitoba, Winnipeg, Manitoba, Canada; 2 Department of Community Health Sciences, University of Manitoba, Winnipeg, Manitoba, Canada; 3 Department of Clinical Health Psychology, University of Manitoba, Winnipeg, Manitoba, Canada; 4 Department of Epidemiology, Netherlands Institute of Mental Health and Addiction, Winnipeg, Manitoba, Canada; 5 Department of Internal Medicine, University of Manitoba, Winnipeg, Manitoba, Canada; 6 Department of Community Health Science, University of Calgary, Alberta, Canada; 7 Department of Psychiatry, University of Calgary, Alberta, Canada

**Keywords:** military, common mental disorders, methods, armed forces, longitudinal study, epidemiology

One of the strengths of research using a longitudinal design is its ability to compare individuals over time. However, attrition from follow-up threatens the validity of such comparisons. If individuals who fail to respond to a questionnaire over time are systematically different from individuals who do respond, estimates of trends may be biased.^1^ A limited body of research has examined longitudinal attrition specific to military populations. Of those published, many of the characteristics associated with loss to follow-up overlap with those noted in nonmilitary samples, including male sex, lower educational attainment, and younger age,^2^ while military-specific correlates include lower rank and military separation.^3,4^ However, a dearth of information exists on psychiatric correlates of attrition, particularly in the military context.

This Special Issue of the *Canadian Journal of Psychiatry* highlights findings from the 2018 Canadian Armed Forces and Veterans Mental Health Follow-up Survey (CAFVMHS). The CAFVMHS is a 16-year follow-up of a representative sample of 5,155 active-duty Canadian Armed Forces personnel from the Canadian Community Health Survey–Canadian Forces Supplement (CCHS-CFS) collected in 2002. An examination of baseline correlates in relation to attrition in CAFVMHS is required.

**Figure 1.**
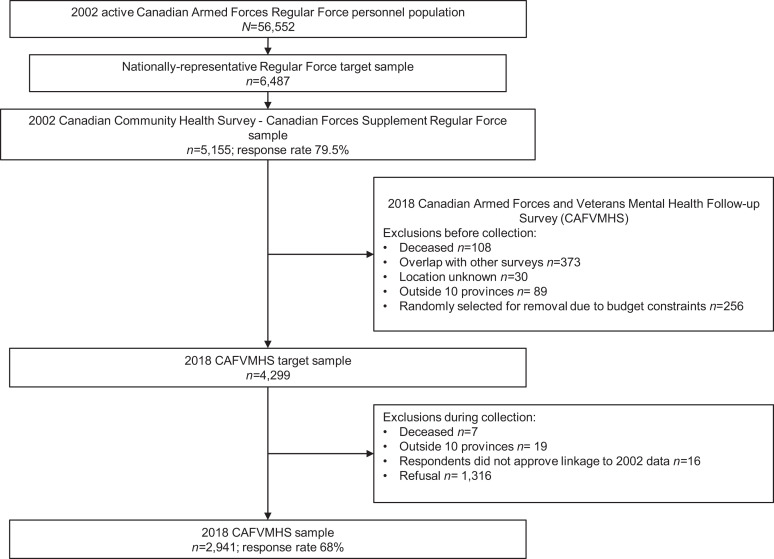
.Composition of the 2018 Canadian Armed Forces Members and Veterans Mental Health Follow-up Survey

To this end, we evaluated attrition in two ways: (1) completion versus noncompletion of 2018 survey; and (2) completion versus three reasons for attrition: (1) deceased: those determined to be deceased either before or during data collection, (2) refused: those who did not respond to requests for follow-up and those who responded to the 2018 CAFVMHS but did not agree to link their data to the baseline data set, and (3) excluded: individuals residing outside the 10 provinces of Canada, those who had already been interviewed for another Statistics Canada military survey (i.e., 2016 Life After Service Survey or the 2016 Canadian Armed Forces Transition and Well-being Survey), those who could not be traced, and those who were removed at random due to budgetary constraints before collection. Figure 1 illustrates the exclusions and composition of the CAFVMHS sample.

Data were analyzed using STATA MP 16^5^ and were unweighted to evaluate actual differences in cells across attrition categories. Potential correlates were based on information recorded at baseline, including sociodemographic and military demographic factors, deployment history, lifetime traumatic exposures, adverse childhood events, and history of mental disorders or suicidality. Cross-tabulations were performed to determine the prevalence of each correlate by attrition type. Unadjusted and adjusted bivariate and multinomial logistic regression models were used to test whether significant (at *P* < 0.05) differences existed in correlates of attrition in reference to follow-up completers.

Of the 5,155 persons included in the CCHS-CFS at baseline, 2,214 (42.9%) did not participate in the CAFVMHS follow-up. A more detailed examination of the sample by reasons for attrition revealed that 115 (2.2%) were deceased, 1,332 (25.8%) refused, and 767 (14.9%) were determined to be ineligible and were excluded from the sample. The total attrition rate in CAFVMHS among those eligible to participate was 31.2%, which is comparatively lower than rates found in general population studies with a similar follow-up period.^2^

Baseline predictors of overall attrition included younger age (odds ratio [OR] = 0.74, 95% confidence interval [CI]: 0.64 to 0.84 for >41 years) and being a member of the Canadian Army (i.e., ground force; OR = 1.19, 95% CI, 1.05 to 1.35). Traumatic exposures, deployment history, childhood adversity, and lifetime psychiatric diagnoses were not found to be determinants of attrition. However, a lifetime history of suicide attempt at baseline significantly correlated with greater overall attrition (OR = 1.42, 95% CI, 1.02 to 1.97).

When examining specific categories of attrition, older individuals (35+) and those in higher ranks (i.e., officers and senior noncommissioned members) were significantly less likely to refuse to participate in the follow-up (ORs ranging from 0.61 to 0.83). Females, those who were separated/widowed/divorced, and officers were more likely to have been excluded from sampling (ORs ranging from 1.27 to 1.41). Representing 5.2% of those who were not reinterviewed, decedents were more likely to be male (OR = 0.63, 95% CI, 0.39 to 0.99 for females), over the age of 40 at baseline (OR = 2.40, 95% CI, 1.50 to 3.82), and to be separated/widowed/divorced (OR = 1.34, 95% CI, 1.03 to 1.74). A history of suicide attempt was linked with both a refusal to participate and attrition due to death (cell sizes too small to be reported).

In sum, only a few demographic characteristics were associated with loss to follow-up. Military status, common mental disorders, traumatic experiences, and childhood adversities were not associated with attrition. These findings align with prior evaluations of attrition in military and nonmilitary longitudinal studies,^2–4^ and expand our understanding to a Canadian military context. Although it has been suggested that attrition in longitudinal psychiatric epidemiologic studies is related to mental health issues among participants, this did not appear to be the case in this sample. Knowledge of this evaluation is vital to interpretation of risk estimates in the longitudinal context among Canadian military personnel and, more specifically, when using the CAFVMHS data.
